# Quality and Brands of Amoxicillin Formulations in Nairobi, Kenya

**DOI:** 10.1155/2020/7091278

**Published:** 2020-06-12

**Authors:** Lilian C. Koech, Beatrice N. Irungu, Margaret M. Ng'ang'a, Joyce M. Ondicho, Lucia K. Keter

**Affiliations:** ^1^Department of Chemistry, School of Pure and Applied Sciences, Kenyatta University, P.O. Box 43844-00100, Nairobi, Kenya; ^2^Center for Traditional Medicine and Drug Research, Kenya Medical Research Institute, P.O. Box 54840-00200, Nairobi, Kenya

## Abstract

Antibiotics are among the most counterfeited anti-infectious medicines in developing countries. Amoxicillin is one of the commonly prescribed, affordable, and easily accessible antibiotic in Kenya. It is a broad-spectrum antibiotic hence commonly used in chemotherapy. This study sought to determine the quality and identify the various brands of amoxicillin and its combination amoxicillin/clavulanic acid marketed in Nairobi County. Nairobi is the capital city of Kenya, gateway for imports and exports, and the headquarters to most of the pharmaceutical distributors. Ten wards in Nairobi County representing different socioeconomic settings were purposively sampled for the study. A detailed questionnaire was used to collect background data on brands of amoxicillin and amoxicillin/clavulanic acid in the market. A total of 106 different brands were found in the market: 85 were imports while 21 were locally manufactured. Fifty-three samples were analyzed with reference to the United States Pharmacopoeia. Amoxicillin and clavulanic acid contents for oral suspensions were determined immediately after reconstitution and 7 days thereafter to determine their stability during the prescription period. On day seven, 23.1% (3 out of 13) of amoxicillin and 66.7% (8 out of 12) amoxicillin/clavulanic acid oral suspensions presented levels below recommended limits. Uniformity of weight for amoxicillin capsules noted 13.6% (3 out of 22) failure rate, while amoxicillin/clavulanic acid tablets complied. Potency determination for all amoxicillin capsules analyzed were within required limits, but amoxicillin/clavulanic acid tablets showed 33.3% (2 out of 6) noncompliance. For amoxicillin capsule and amoxicillin/clavulanic acid tablet dissolution tests, there was 10.5% (2 out of 19) and 50% (2 out of 4) noncompliance, respectively. Overall, 37.7% of the drugs analyzed failed to comply with the Pharmacopoeia. These results highlight the presence of poor-quality amoxicillin formulations in Nairobi County, affirming the need for regular postmarket surveillance to inform on the situation of antibiotic quality in the Kenyan market.

## 1. Introduction

Over the past decade, increased public awareness on drug quality has been assessed in terms of counterfeit and/or substandard products [1]. According to the United States Food and Drug Administration (FDA), 50% of medicines being sold in Africa are thought to be counterfeit and/or substandard [2, 3]. Studies have reported anti-infectious agents, particularly antibiotics and antiparasitics, being highly predisposed to compromised quality especially in developing countries due to their high demand [4, 5]. Orally administered anti-infective formulations accounted for 77%; thus, they are the most counterfeited antibiotic medicines [6]. Efforts to conduct studies on the quality of drugs in the Kenyan market have been reported since the early 1980s. According to reports by the Kenya National Quality Control Laboratory (NQCL), the failure rate for antibiotics was reported at 24.3% in the year 1996-2001, 9.4% in 2004-2005, and 17.3% in 2006-2007 [7].

Amoxicillin is an antibiotic that belongs to a class of drugs called penicillin, which fall in the beta-lactam family of antibiotics [8]. *β*-Lactam utilization accounts for 65% of the total global antibiotic market [9], because they work extremely well against a wide variety of bacteria while exerting little toxicity towards mammalian cells [10]. Amoxicillin is predisposed to degradation by beta-lactamase-producing bacteria; thus, it may be administered with clavulanic acid, a *β*-lactamase inhibitor [11]. The combination is used as a broad-spectrum antibiotic for the treatment of a wide range of bacterial infections, including upper and lower respiratory tract infections and infections of the skin and soft tissue [12].

In Kenya, the magnitude of substandard and/or counterfeit antibiotics is unknown, yet the issue of poor-quality drugs is extensively discussed in the media and respective ministries ([13–15]; [16–19]). There have been few studies in which the quality of amoxicillin has been analyzed in Kenya [20–22] all of which reported the presence of poor quality but none in a scope that informs on the situation in our Kenyan market. As a consequence, there is a need for information on the quality of such commonly prescribed antibiotics to create awareness for public information. For that reason, the aim of this study was to identify the various brands and determine the quality of amoxicillin formulations marketed to the public within Nairobi County.

## 2. Materials and Methods

### 2.1. Study Location

**Figure 1:**
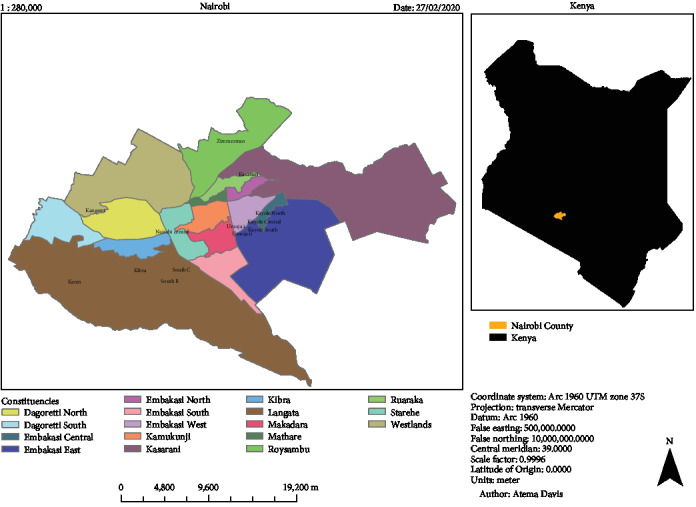
Nairobi County map showing the location of the selected wards [26].

This study was undertaken in Nairobi County, the capital city of Kenya and home to the largest airport in East Africa serving as the gateway for imports and exports, thus making it the commercial hub of the country. In addition, it is the headquarters of most of the pharmaceutical distributors and wholesalers in the country [23, 24]. Nairobi has seventeen subcounties which are further divided into eighty-five wards. It has a population of about 3 million people contributing to 8.1% of the Kenyan population, hence it is the most populous county in the country [25].  Figure 1 displays the Nairobi County map with the study wards indicated.

### 2.2. Study Design

This was a cross-sectional study to identify brands and quality of amoxicillin formulations from retail and private hospital pharmacies in different locations within the county. The locations were selected based on economic stratification: upper-, middle-, and lower-income classes [27, 28]. The wards with an upper-income population included Westlands/Kangemi and Karen; middle-income population wards were South B and South C; middle–lower-income populations included Zimmerman and Kasarani; while lower-income population wards were Kibra, Kayole, and Umoja (Figure 1).

A market surveillance was first undertaken in the 10 wards in the county to identify the various brands of amoxicillin and amoxicillin/clavulanic acid drugs in March and April of 2016. This was achieved by use of a data capture form with open-ended questions administered by trained research assistants. Additional information such as manufacturer and country of origin were also captured. Personnel-in-charge of the facility were informed of the nature of the study and assured of confidentiality before data collection or drug sampling.

According to the Pharmacy and Poisons Board-Kenya register of 2015, 998 pharmacies were registered in Nairobi (http://www.Pharmacyboardkenya.org/?p=530). Using the Krejcie and Morgan sample table [29], 284 retail and private hospital pharmacies were visited to identify the various brands of amoxicillin and amoxicillin/clavulanic acid drugs but only 278 responded (98% response rate).

Thereafter, samples were purchased for analysis from 168 out of 177 (95% response rate) retail and private hospital pharmacies located in these wards selected through purposive sampling. In each retail and private hospital pharmacies sampled, all the amoxicillin and amoxicillin/clavulanic acid brands stocked were sampled to eliminate bias. A total of 148 samples were sampled between April and July 2016 which formed the primary samples. Subsequently, secondary sampling was carried out purposively by eliminating brands with similar batch numbers from the same wards, while unique brands were given priority to achieve a sample size of 53. These consisted of 6 amoxicillin/clavulanic acid tablets, 22 amoxicillin capsules, and 13 amoxicillin and 12 amoxicillin/clavulanic acid dry oral suspensions. Two controls of the innovator drugs, an amoxicillin/clavulanic acid tablet with a strength of 625 mg and an amoxicillin oral dry suspension with a strength of 250 mg/5 ml, were sourced from one of the official local distributors. The samples were assigned unique codes based on study location.

### 2.3. Chemicals and Reagents

Amoxicillin trihydrate standard (*w*/*w* 99.95%) and clavulanate potassium standard (92.75%) from Medopharm Private Limited (Guduvanchery, India) were generous gifts from Dawa Limited and National Quality Control Laboratory (NQCL), respectively. Potassium dihydrogen phosphate (Sigma Aldrich Chemical Co. Ltd., Gillingham, Dorset, UK) and phosphoric acid (Merck Pvt. Ltd., Guateng, South Africa) were of analytical grade. Acetonitrile (Sigma-Aldrich Co. Steinheim, Germany) and methanol (Thermo Fischer Scientific UK Ltd., Loughborough, UK) were of HPLC grade solvents.

### 2.4. Equipment and Materials

The liquid chromatographic system consisted of an Agilent 1200 Infinity series high-performance liquid chromatography (LC) system (Agilent Technologies, Deutschland, Germany) supported by OpenLAB software version A.01.03 and equipped with a G1314B Agilent 1260 Infinity Variable Wavelength UV Detector. The temperature was controlled using a G1316A column oven with a G1330B Agilent 1260 Infinity Thermostatted Column Compartment. The LC system also had a G1311C Agilent 1260 Infinity Quaternary Pump and a G1329B Autosampler. A dissolution tester (DS 800, Lab India, Pvt. Ltd., Mumbai, India) was used in the study. A double-beam T90+ UV/Vis spectrophotometer supported by the UVWIN software version 5.2.0 (PG Instruments Ltd., Leicestershire, United Kingdom) and quartz cuvettes with a path length of 1 cm were used.

A Barnstead Smart2Pure™ water purification system (Thermo Fisher Scientific, Massachusetts, United States) was used to obtain ultrapure water. A Shimadzu AUW220D semi-micro-analytical electronic weighing balance (Shimadzu Corporation, Kyoto, Japan) with a sensitivity of ±0.1 mg was used for weighing. All the mobile phase preparations were degassed using a MRC DC-200H Ultrasonic Cleaner (MRC Lab Ltd., Holon, Israel).

### 2.5. Determination of Uniformity of Weight

Uniformity of weight determination was carried out for 28 solid dosage preparations (tablets and capsules samples) plus a control to check for consistency in weight of dosage units. Twenty units from each of the solid dosage formulations and the control were taken at random and then weighed. The content of each of the 20 weighed tablets and capsules from the respective samples were separately homogenized, labelled, and stored in air-tight amber containers for content determination. Average weights and percentage deviations from the mean values were calculated for each sample and control.

### 2.6. Potency Determination

The determination of amoxicillin and clavulanic potency was performed according to criteria established by the USP [30]. The percentage label claim (% L.C.) of each drug sample was obtained by comparing the average peak areas and concentrations of both the standard and sample solutions putting into account the average weight in case of tablet or capsule contents, the purity of the standard, and the label claim contents of each sample.

#### 2.6.1. System Suitability Test (SST)

System suitability test was performed routinely before sample analysis could commence. It is undertaken to verify resolution, column efficiency and repeatability of a chromatographic system to guarantee its competence for a particular analysis [31]. Content assay of the active pharmaceutical ingredients (API), dissolution testing, and impurity determinations must pass a set of predefined acceptance criteria (Table 1), hence an integral part of any analytical procedure [32].

#### 2.6.2. Mobile Phase System

The mobile phase solution for amoxicillin capsules and oral dry suspensions consisted of acetonitrile and 0.05 moles potassium dihydrogen phosphate buffer pH 5.0 (1 : 24, *v*/*v*). The pH was adjusted using potassium hydroxide solution (45% *w*/*w*). The mobile phase for amoxicillin/clavulanic acid tablets and oral suspensions consisted of acetonitrile and 0.057 moles potassium dihydrogen phosphate buffer pH 5.0 (1 : 19, *v*/*v*). The pH was adjusted using phosphoric acid. The buffers were filtered through 0.45 *μ*m nylon filters and degassed before use.

#### 2.6.3. Amoxicillin Capsule Sample Preparation

The homogenized contents of each sample stored in air tight amber containers were weighed. A quantity of about the average weight obtained from a uniformity of weight test done earlier was weighed accurately in triplicates for each sample. Only samples that complied with the compendia specification for uniformity of weight were subjected to API determination.

#### 2.6.4. API Determination

The samples were either in 250 mg or 500 mg strength. Each sample was weighed into 200 ml volumetric flasks and topped to mark with the buffer. The solutions were sonicated for 10 minutes. Aliquots of 20 ml for 250 mg and 10 ml for 500 mg of the resulting solutions were pipetted into 25 ml volumetric flasks and made to mark with the buffer, obtaining the concentration of approximately 1 mg/ml. The solution was filtered through a 0.45 *μ*m syringe-adaptable membrane filter into LC amber vials and analyzed. Each of the replicates was injected three times.

#### 2.6.5. Amoxicillin Dry Oral Suspension Sample Preparation

A total of thirteen samples with strengths of 125 mg/5 ml and 250 mg/5 ml and control (250 mg/5 ml) were reconstituted with ultrapure water to the mark as specified on the labeling and thoroughly mixed by shaking. Aliquots of 8 ml (125 mg/5 ml) and 4 ml (250 mg/5 ml) were accurately taken in three replicates using analytical volumetric pipettes into 200 ml volumetric flasks and topped up to volume with the mobile phase. The solutions were sonicated for 10 minutes; obtaining the concentration of approximately 1 mg/ml. The solutions were filtered using a 0.45 *μ*m membrane filter into LC amber vials and analyzed. Each of the replicates was injected three times. The samples and control were analyzed on the day they were reconstituted (day zero) and on the seventh day after reconstitution to determine their stability. The samples were stored at room temperature (25 ± 1°C) after reconstitution.

#### 2.6.6. Amoxicillin Standard Preparation

Amoxicillin standard was prepared as per the compendia method [30]. Approximately 12 mg of amoxicillin trihydrate with a standard potency of 99.95% was accurately weighed in two replicates A and B into a 10 ml volumetric flasks, dissolved and made to volume with the buffer solution, obtaining the concentration of about 1.2 mg/ml. The solution was sonicated for 7 minutes and filtered through a 0.45 *μ*m membrane filter into LC amber vials and analyzed. Each of the replicates was injected three times.

#### 2.6.7. Amoxicillin/Clavulanic Acid Tablet Sample Preparation

Twenty tablets of each of the 6 samples of tablets and the control were crushed into fine powder with a pestle and mortar. The weight equivalent to the average weight for each sample were accurately weighed. The tablets had strengths of 375 mg and 625 mg and control (625 mg). Each sample and control were weighed in three replicates into 100 ml volumetric flasks, dissolved in and made to volume with the buffer. The solutions were sonicated for 10 minutes. Aliquots of 7 ml (375 mg) and 4 ml (625 mg) of the resulting solutions were pipetted into 50 ml volumetric flasks and made to the mark with the buffer, obtaining a concentration of approximately 0.5 mg/ml. The solutions were filtered through a 0.45 *μ*m membrane filter into LC amber vials and analyzed. Each of the replicates was injected three times.

#### 2.6.8. Amoxicillin/Clavulanic Acid Dry Oral Suspension Sample Preparation

Twelve samples of amoxicillin/clavulanic acid dry oral suspension samples (with strengths of 228.5 mg/5 ml, 457 mg/5 ml, and 642.9 mg/5 ml) were all reconstituted with ultrapure water to the mark as specified in the labeling, thoroughly mixed, and freed of air bubbles. Three replicate volumes of 4 ml (642.9 mg/5 ml) were then taken using analytical volumetric pipettes into 200 ml volumetric flasks and accurately topped up with the buffer. The solution was sonicated for 10 minutes. This made a stock solution from which aliquots of 4 ml were transferred into a 20 ml volumetric flask and made to mark with the buffer to make the final concentration of approximately 0.5 mg/ml. The same procedure was repeated for 228.5 mg/5 ml and 457 mg/5 ml where three replicate aliquots of 3 ml each were transferred into 100 ml and 200 ml volumetric flasks, respectively. This made a stock from which 4 ml was taken into a 10 ml volumetric flask to make the final concentration of about 0.5 mg/ml. The samples were analyzed on the day they were reconstituted and on day 7 after reconstitution to determine their stability. The solution was filtered through a 0.45 *μ*m membrane filter into LC amber vials and analyzed. Each of the replicates was injected three times. The samples were stored at room temperature (25 ± 1°C) after reconstitution.

#### 2.6.9. Amoxicillin and Clavulanic Acid Standard Preparation

As per the monograph [30], approximately 25 mg of amoxicillin trihydrate with a standard potency of 99.95% and 10 mg of clavulanate lithium with a potency of 96.4% were weighed (mixed) in two replicates A and B to a 50 ml volumetric flask. This was dissolved in ultrapure water and topped up to the 50 ml mark to produce a final concentration of approximately 0.5 mg/ml of amoxicillin and 0.2 mg/ml of clavulanate lithium. The solution was filtered through a 0.45 *μ*m membrane filter into LC amber vials and analyzed. Each of the replicates was injected three times.

#### 2.6.10. Chromatographic System for Content Determination

The chromatograph wavelength was set at 220 nm and separation was achieved from Symmetry® C18, 5 *μ*m, 250 × 4 mm column (Waters Corp., Massachusetts, USA) maintained at 40 ± 1°C in a thermostat oven. The injection volume was set at 10.0 *μ*l and the flow rate at 1.50 ml/min.

### 2.7. Dissolution Studies

Dissolution studies were undertaken for solid dosage preparations according to the USP [30]. Dissolution station apparatus was set to 37°C ± 0.5°C for 30 minutes and a frequency of 75 rotations per minute (rpm). A set volume of 900 ml of dissolution medium (distilled water) was accurately measured using a 1000 ml measuring cylinder and poured into each of the six glass vessels and maintained at the set temperature. Standard thermometers were placed in each vessel to crosscheck the temperature. Only samples that complied with the standard requirement for API were subjected to the dissolution studies. These comprised of 19 samples of amoxicillin capsules, 4 samples of amoxicillin/clavulanic acid tablets, and the control. Six from every sample and control were placed into the six dissolution glass vessels. At the end of the run time, aliquots of 20 ml were taken from each vessel. The drug solutions were allowed to equilibrate to room temperature and portions filtered.

Amoxicillin/clavulanic acid tablet aliquots were transferred into amber vials and analyzed using LC with Symmetry® C18, 250 × 4 mm, 5 *μ*m column (Waters Corp., Massachusetts, USA) maintained at 40°C in a thermostat oven; wavelength was set at 220 nm. The mobile phase consisted of acetonitrile and potassium hydrogen phosphate buffer pH 5.0 (1 : 24 *v*/*v*) at a flow rate of 2.0 ml/min. The injection volumes were 20 *μ*l and were made in triplicates.

For amoxicillin samples, the aliquots obtained formed the stock solution from which 10 ml (for 250 mg sample strength) and 5 ml (for 500 mg sample strength) for amoxicillin capsules were accurately transferred into a 25 ml volumetric flask and made to mark with ultrapure water and analyzed using a UV/Vis spectrophotometer at 272 nm against amoxicillin trihydrate standard with a concentration of 0.1 mg/ml.

### 2.8. Statistical Analysis

All results were recorded as the mean values ± SD of at least three replicates for each by using Statistical Package for Social Scientists (SPSS) software version 21.0 and Excel MS 2016.

### 2.9. Ethical Approval

Ethical approval was obtained from the Kenya Medical Research Institute Scientific and Ethics Review Committee (SERU) (KEMRI/SERU/CTMDR/012/3059). Amoxicillin and amoxicillin/clavulanic acid are prescription-only drugs, hence an authorization letter from the Kenya Medical Research Institute allowing for the purchase of the samples was presented.

## 3. Results

### 3.1. Brand Frequency and Countries of Origin of Amoxicillin and Amoxicillin/Clavulanic Acid in the Market

**Figure 2:**
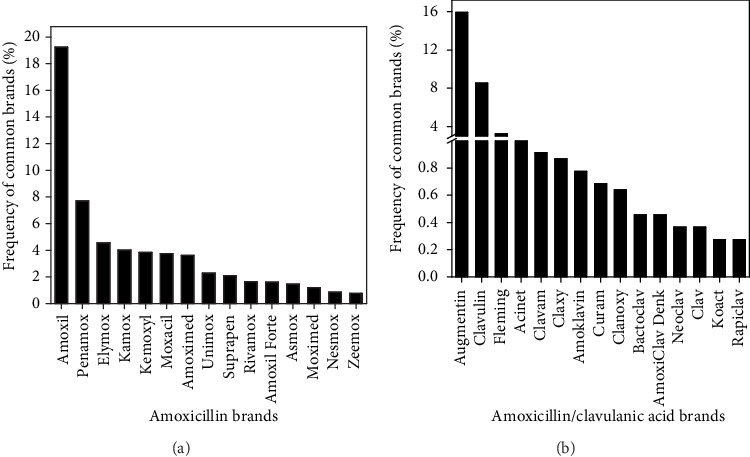
Predominant brands of amoxicillin (a) and amoxicillin/clavulanic acid (b) drugs stocked within Nairobi County.

Out of 278 pharmacies that responded, 99.6% (277) stocked amoxicillin and amoxicillin/clavulanic acid products. A total of 106 different brands were found in the market, among which 52 were single-molecule amoxicillin imports while 33 were amoxicillin/clavulanic acid combination imports. Locally manufactured brands accounted for 21 of the brands recorded and were all single-molecule amoxicillin products. This study further established that all (100%) of the amoxicillin/clavulanic acid drugs found in Nairobi market were imported. More than half (72.6%) of the pharmacies visited stocked the innovator brands for both single-molecule formulations (Amoxil®) and amoxicillin/clavulanic acid combination (Augmentin®). Amoxil® accounted for 19.3% of all amoxicillin samples recorded in the sampled locations while Augmentin® was 15.9% as shown in Figure 2. Innovator brands Amoxil® and Augmentin® were predominantly stocked in Nairobi Central (28.2%), a location that serves all income classes and in the high- and middle-income areas; 14.4% being in Westlands, 10.4% in Karen, 7.7% in South C, and 9.3% in South B. In lower-income populations, 4.5% were stocked in Kibra and 6.2% in Kayole, while in middle–lower-income populations, 6.0% were in Zimmerman and 7.2% in Kasarani. The most commonly stocked generics for amoxicillin and amoxicillin/clavulanic acid formulations were Penamox® (7.7%) and Clavulin® (8.5%), respectively.

**Figure 3:**
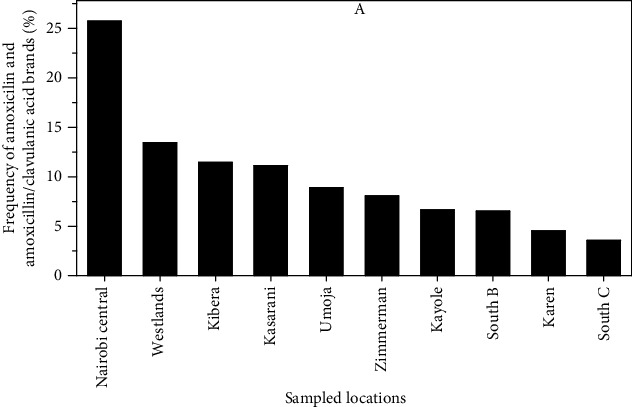
Frequency of amoxicillin and amoxicillin/clavulanic acid brands available in sampled wards within Nairobi County, Kenya.

Generally, Nairobi Central had the highest frequency of amoxicillin drugs stocked in pharmacies at 25.7% as shown in Figure 3, followed by Westlands at 13.5%. Pharmacies in the South C area had the lowest number of amoxicillin products stocked at 3.6%.

Majority (85.2%) of amoxicillin brands were imports with 43.9% being imported generic products, while 41.3% were innovator imported brands (Amoxil® and Augmentin®). Innovator brands were mainly from the United Kingdom (25.5%), France (8.6%), and Belgium (6.9%), while generic import products were predominantly from India (27.4%), Canada (6.4%), and China (2.4%). Others products were from Egypt (2.2%), United States (1.5%), Pakistan (1.0%), Germany (0.55%), Turkey (0.7%), Austria (0.4%), Mexico (0.32%), South Africa (0.3%), Lebanon (0.14%), Thailand (0.14%), Indonesia (0.14%), Philippines (0.09%), Malaysia (0.09%), Switzerland (0.05%), Cyprus (0.05%), Algeria (0.05%), and Argentina (0.05%). Locally manufactured amoxicillin products accounted for 14.8% of the brands found in the market.

### 3.2. Uniformity of Weight Determination

All the amoxicillin/clavulanic acid samples and control were within the acceptable uniformity of weight limits [32] as shown in Table 2.

Table 3 shows that 86.4% (19 out of 22) of the amoxicillin capsule samples complied with the USP compendia specifications for uniformity of weight [32]. According to the USP specifications, not more than two of the individual masses should deviate from the average mass of the percentage weight deviation, and none should deviate more than twice that percentage weight [32]. It was noted that the samples that failed were imported products and were eliminated from assay determination and dissolution tests.

### 3.3. Potency Determination for Oral Dry Suspensions

#### 3.3.1. System Suitability Parameters (SST) and Active Ingredient Identification

The chromatographic system parameters were within the USP limits as given in Table 1.

**Figure 4:**
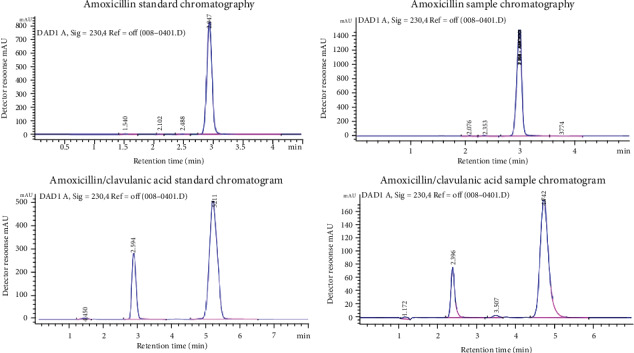
Typical chromatograms: (a) amoxicillin standard chromatography, (b) amoxicillin sample chromatography, (c) amoxicillin/clavulanic acid standard chromatogram, (d) amoxicillin/clavulanic acid sample chromatogram. Chromatographic parameters: C18 4.6 *μ*m column; temperature 40°C; injection volume 10 *μ*l; flow rate 1.5 ml/min.

Active ingredient identification test was first undertaken to ascertain that the samples contained the targeted API using HPLC as per the USP method [30]. The retention times of major peaks of all amoxicillin and amoxicillin/clavulanic acid samples corresponded to that of the reference standards. The retention time for amoxicillin samples and the standard was at 2.8 ± 0.2 minutes. Similarly, that of combination molecule; amoxicillin was at 2.7 ± 0.2 minutes, while that of clavulanic acid was 5.0 ± 0.2 minutes (Figure 4).

#### 3.3.2. Amoxicillin Oral Dry Suspension Potency Determination

On day zero, 92.0% (12 out of 13) of analyzed amoxicillin oral suspension samples met the specified USP limits for content, while on day seven, 85.0% (11 out of 13) of the samples and the control were still within the USP limits. However, 23.1% (3 out of 13) failed to meet the USP limits for assay as presented in Table 4 and were all imported generic products. They were sampled from South B, South C, and Kibra.

#### 3.3.3. Amoxicillin/Clavulanic Acid Oral Dry Suspension Potency Determination

Immediately after reconstitution (day zero), 75.0% (9 out of 12) of the samples analyzed complied with USP specifications for assay as indicated in Table 5. All the samples were within compendia limits for clavulanic acid content, while 25.0% (3 out of 12) of the samples did not meet the limits for amoxicillin content. On day seven, only 33.0% (4 out of 12) of the samples were within specifications for content assay for both active ingredients. Half of the samples (50.0%; 6 out of 12) were below pharmacopoeial limits for amoxicillin content. Eight out of twelve (66.7%) samples failed to meet pharmacopoeial specifications and were imported products. Three were sampled from Nairobi Central, 2 from South C, and 3 each from Kibra, Weslands, and Kayole wards. In general, only 56.0% (14 out of 25) of the samples were within the compendia limits for content analysis for both single- and combined-dosage formulation samples.

### 3.4. Potency Determination and Dissolution Test for Tablets and Capsules

#### 3.4.1. Amoxicillin Capsules

All 19 amoxicillin capsule samples analyzed met the specified limits for chemical content while seventeen of nineteen (89.5%) complied with the pharmacopoeial specifications for dissolution test as shown in Table 6. Dissolution chemical contents of 2 imported samples were outside the required limits and were sampled from Kayole and South C wards.

#### 3.4.2. Amoxicillin/Clavulanic Acid Tablets

The control and 66.7% (4 out of 6) of amoxicillin/clavulanic acid samples analyzed were within the specifications for API content as shown in Table 7. All the samples were within limits for clavulanic acid content while 2 imported samples, each sampled from Nairobi Central and South B wards, did not meet specifications for amoxicillin content, hence they were eliminated from dissolution studies. Only 4 samples and the control were therefore subjected to a dissolution test and only 2 (50%) met the compendia requirements for dissolution as shown in Table 7.

#### 3.4.3. Summary of Sample Quality from the Same Manufacturer

When comparing samples from the same manufacturers but with different batch numbers, varying results were noted as shown in Table 8.

## 4. Discussion

The magnitude of counterfeit or substandard antibiotics in Kenya has not yet been quantified [19, 33]. However, good quality medicines are essential in management and treatment of diseases and contribute to the achievement of universal health coverage (UHC). This study established that amoxicillin was commonly stocked in pharmacies within Nairobi County. This concurs with the WHO Expert Committee [34] report on essential medicines that recommends amoxicillin as a drug with lower potential for resistance and should be readily accessible for the treatment of a wide range of common bacterial infections. In addition, innovator and generic amoxicillin drugs in the Kenyan market are largely imports at 85.2%. Further noted was that all the locally manufactured drugs (14.8%) in the market were a single molecule of amoxicillin while all the combination clavulanic/amoxicillin acid products (100%) were imports. A report on prices and availability of medicines in the Kenyan market similarly noted that predominantly imported generics were from India and China [23]. A total of 106 different brands were found in the market of which only 21 (19.8%) were locally manufactured; however, there could be more brands that were not captured. Studies have shown that having too many brands of the same molecule poses challenges in ensuring quality to the regulatory body [23, 35]. Most of the pharmacies stocked (99.6%) innovator brands of single-molecule amoxicillin (Amoxil®) and its combination amoxicillin and clavulanic acid (Augmentin®). Comparable findings were reported in a survey on the medicine pricing in Kenya which noted that innovator brands were mostly sold in Nairobi, with availability being twice more likely than in other regions in Kenya [36]. Nairobi Central and Westlands wards had the highest stocked brands of both innovator and generic products (Figure 3). According to the Ministry of Health [37], these two wards were reported to host most pharmaceutical distributor outlets and pharmacies.

Uniformity of weight was employed as a good pointer to Good Manufacturing Practice (GMP). This assures the drug content in each unit dose is evenly distributed in a normal range around the labelled active ingredient. Weight variation vividly reflects on the content of the active ingredient [38]. All the amoxicillin/clavulanic acid tablet samples met the compendia specification for uniformity of weight (Table 2). These results agreed with a previous study done in Ethiopia [39].

Amoxicillin capsules, however, recorded a failure rate of 13.6% as shown in Table 3. Related results were reported in a study done in Nigeria on amoxicillin capsules in which 30% of the samples analyzed failed to comply [40]. Once such weight variances are observed, it is thereafter impossible to assure the active ingredient content of the drug units [41, 42]. It is for this reason that the brands which failed the weight uniformity test were eliminated from subsequent tests.

All samples analyzed displayed retention times orresponding with that of the respective standards. This confirmed that all samples contained the targeted API [43].

Potency determination is critical for quality assessment of active pharmaceutical content. Appropriate content is important in attaining effective concentration at the site of infections when administered [44, 45]. Oral suspensions of amoxicillin are available in the market as dry powders for reconstitution and should be refrigerated thereafter to prevent degradation of active ingredients. However, most households do not own refrigerators and hence store these drugs at room temperature. Studies have reported that the penetration level of refrigeration appliances in Nairobi is at 35% majorly in high- and middle-income level households [46]. Dry oral suspensions should be within the recommended compendia specifications immediately after reconstitution and seven days after for both amoxicillin and clavulanic acid contents [32]. Substantial variations could result in ineffective or toxic therapeutic drug levels [47].

Out of the thirteen amoxicillin dry oral suspension samples analyzed, 23.1% presented values below compendia requirements on the seventh day (Table 4). This is consistent with the findings of Taylor et al. [48] that reported low API quantities in 40% of amoxicillin oral dry suspensions analyzed. Similar results were also documented in Saudi Arabia where 8% on day zero and 38% on day seven of amoxicillin oral dry suspensions analyzed were below pharmacopoeial limits [49].

Twenty-five percent of amoxicillin/clavulanic acid oral dry suspensions were below compendia limits for amoxicillin on day zero but were within the limits for clavulanic acid (Table 5), while by day seven an additional 5 samples were below pharmacopoeial specification limits [30] resulting in a 66.7% (8 out of 12) failure rate. This shows lack of stability of the drugs over the prescription period as significant degradation was noted by the seventh day of use especially for the amoxicillin and clavulanic acid combination. This implies that the patient will receive suboptimal dosages during the latter treatment stages thus resulting to induction of resistance in the target bacteria or host toxicity from degradation products [50]. Nettey et al. [51] reported comparable findings in Ghana with amoxicillin content showing more failure than clavulanic acid.

All the nineteen amoxicillin capsule samples met the pharmacopoeial specifications for potency determination (Table 6) [30]. Related results were reported in Ethiopia [52] and in a study on quality of antibiotics in Ghana, Nigeria, and the United Kingdom where 95% of amoxicillin capsules analyzed complied with the specified tolerance limits [53]. On the contrary, a study conducted in India on generic amoxicillin capsules bought from open market vendors recorded 28.3% of the products outside the specifications [54]. Nguyo et al., (2013) similarly reported 50.0% noncompliance of locally manufactured amoxicillin capsules analyzed from 2006 to 2010.

In this study, 66.7% of amoxicillin/clavulanic acid tablets investigated were within the specifications for API content (Table 7). Contrasting results were stated in Ghana where all amoxicillin/clavulanic acid samples analyzed were outside required limits [51], while in Nigeria and Ethiopia, Olanrewaju et al. [55] and Mekonnen et al. [39], respectively, reported compliance with compendia limits for all amoxicillin/clavulanic acid tablet samples investigated. Many factors such as storage conditions, transportation during distribution, components of drug composition, and nature of the active ingredient used during formulation may affect the content of a drug [4, 56, 57].

Dissolution is the primary quality control test to determine whether a drug product can release its active pharmaceutical ingredient(s) in a timely manner. It is associated to drug bioavailability and thus drug absorption [58, 59]. It is therefore used to assess batch-to-batch quality and provide process control and quality assurance under optimal operating conditions. Hence, significant variations in the dissolution rate among the same products indicate inconsistencies in the drug formulation and delivery process [60]. According to the USP, the amount of amoxicillin released within 60 minutes for amoxicillin capsules and 30 minutes for amoxicillin/clavulanic acid tablets should not be less than 80% and 85%, respectively, while that of clavulanic acid should not be less than 80% of the labelled claim [30]. Majority (89.5%) of the amoxicillin capsules investigated complied with pharmacopoeial specifications (Table 6). Contrary results were observed in Ghana, Nigeria, and United Kingdom, where Kaur et al. [53] reported compliance with pharmacopoeial requirements of all amoxicillin capsules tested in a study on antibiotics. Only 50% of amoxicillin/clavulanic acid tablets met the specified limits for dissolution. This was inconsistent with studies done by Olanrewaju et al. [55] in Nigeria that reported 86% compliance and with studies done by Nettey et al. [51] in Ghana that reported 93% of amoxicillin/clavulanic acid tablets tested complied with pharmacopoeial specifications.

The presence of poor-quality amoxicillin products was recorded in 70% (7 out of 10) of wards sampled, with Nairobi Central having the highest percentage. This could be because it hosts the central business district and has the highest number of pharmacies, hence it is easy for poor-quality drugs to infiltrate the market [24]. The Kibra ward similarly recorded a significant percentage of substandard drugs with most pharmacies stocking mainly generic products. Kibra hosts Africa's largest urban slum and most of its dwellers can only afford cheaper drugs and can be easily sold poor-quality drugs by unscrupulous dealers [25].

Overall, 37.7% (20 out of 53) of the samples analyzed were of poor quality as per the USP specifications and were all imported products. Hence, this is an indication that more stringent measures should be taken to ensure that imports comply with quality requirements. Samples from Nairobi Central had the highest substandard prevalence at 11.3%, followed by Kibra and South C at 7.5%, South B and Kayole at 3.8%, and Kangemi and Westlands at 1.9% each. There was no relationship between socio-economical setting and product quality as seven out of the ten wards (70.0%) sampled registered the presence of substandard medicines.

Findings in Table 8 show samples from the same manufacturer having varying quality. This is an indication of non-adherence to GMP by some manufacturers, hence leading to batch-to-batch inconsistency [61]. GMP is part of quality assurance that ensures products are consistently manufactured and controlled to the quality standards appropriate to their intended use and as required by the regulating authority [62]. Inconsistency in manufacturing processes increases risks inherent to pharmaceutical production such as cross-contaminations, poor inprocess controls, inappropriate packaging and labeling, and poor storage and transportation processes. This consequently results in substandard and/or counterfeit pharmaceutical products.

## 5. Limitations

Registration status of the various brands of amoxicillin formulations were not confirmed from the official regulator (PPB). Only identification, content determination, uniformity, and dissolution tests were done. Therefore, “quality” in this study refers only to the uniformity of weight of solid dosages, content analysis, and dissolution of the active ingredient in terms of the ranges specified by the USP. Some samples met compendia limits for content analysis and dissolution, while other samples from the same manufacturers failed. It was assumed that this was due to poor manufacturing practices and did not consider degradation of the samples due to poor storage and transportation processes.

## 6. Conclusion

The results from this study shows that some amoxicillin formulations marketed in Nairobi County are of poor quality. Generally, 37.7% of the samples analyzed failed to meet the pharmacopoeial specifications and were all imported products. This observation goes against the perception that imported compared to locally manufactured medicines in developing countries are of better quality. Poor-quality amoxicillin formulations were documented in 70% (7 out of 10) of the sampled wards, hence there was no correlation between socio-economical setting and drug quality. Therefore, there is a need to extend the study to other counties to inform on the situation of antibiotic quality in the Kenyan market. Strict regulation is also essential to guarantee compliance with good manufacturing practices by pharmaceutical manufacturers. Ensuring availability of quality essential medicines to the public through frequent postmarket surveillance is an important contribution in the fight against antimicrobial resistance and reduction in disease burden through mitigating treatment failures. This will contribute towards the achievement of universal health coverage as a key target under the United Nations sustainable development goals which Kenya hopes to achieve by 2030.

## Figures and Tables

**Table 1:** Method SST compared to acceptance limits parameters as per USP.

Parameter	Actual method suitability parameters		Acceptable criteria (USP)
	Amoxicillin	Clavulanic acid	
Precision/injection repeatability	Indicated in the data for respective samples in the result tables		Relative standard deviation ≤ 2%
Resolution factor (*R*)	2.76 to 3.17		*R* > 1.5
Tailing factor (*T*)	0.89 to 1.52	0.99 to 1.6	*T* ≤ 2
Theoretical plates (*N*)	2017-2470	3970-4480	*N* ≥ 2000 plates

**Table 2:** Uniformity of weight for amoxicillin/clavulanic acid tablet samples.

Sample code	Country of origin	Labelled active ingredients (mg)	Average weight uniformity (mg) ± SD	RSD	Mean weight deviation (%)	Number of tablets outside USP range	USP-acceptable criteria
Control	United Kingdom	625	1083.9 ± 9.11	0.8	-1.7300~1.2800	0	±5%
KAN95	India	375	739.7 ± 9.07	1.2	-1.9880~2.7166	0	±5%
SB36	India	375	784.6 ± 6.69	0.9	-1.7334~1.2236	0	±5%
CBD131	India	625	1091.0 ± 14.84	1.4	-2.9532~2.1429	0	±5%
KBR90	India	625	1023.1 ± 17.89	1.7	-3.0920~2.8506	0	±5%
SB57	India	625	1041.0 ± 12.10	1.2	-3.1586~1.9424	0	±5%
CBD136	India	625	1051.1 ± 11.34	1.1	-2.2771~1.6425	0	±5%

RSD: relative standard deviation, SD: standard deviation.

**Table 3:** Uniformity of weight for amoxicillin capsule samples.

Sample code	Country of origin	Labelled active ingredients (mg)	Average weight uniformity (mg) ± SD	RSD	Mean weight deviation (%)	Number of capsules outside USP range	USP-acceptable criteria
KAY52	China	250	312.8 ± 5.31	1.7	-1.8862~3.1969	0	±7.5%
SC10	Kenya	250	288.7 ± 8.37	2.9	-5.2650~4.8147	0	±7.5%
SB32	India	250	301.9 ± 6.07	2.0	-4.0742~5.2004	0	±7.5%
KAY56	India	500	594.8 ± 5.83	1.0	-2.1520~1.9670	0	±5.0%
KAY51	India	250	309.0 ± 9.96	3.2	-8.2201~6.9579	1	±7.5%
ZIM109	Kenya	250	295.7 ± 5.59	1.9	-3.5171~4.1258	0	±7.5%
CBD138	Kenya	250	336.6 ± 10.84	3.2	-5.7932~4.8128	0	±7.5%
KBR78	China	250	300.0 ± 7.70	2.6	-3.2000~5.000	0	±7.5%
KAN120	United Kingdom	500	588.6 ± 6.58	1.1	-2.3955~1.9198	0	±5.0%
SC13	India	500	579.9 ± 7.75	1.3	-2.5349~2.2935	0	±5.0%
SB21	China	500	585.4 ± 11.17	1.9	-2.6478~4.0656	0	±5.0%
KBR84	Kenya	500	591.7 ± 4.49	0.8	-2.0788~1.1323	0	±5.0%
KBR87	India	250	296.9 ± 6.47	2.2	-4.8501~2.8629	0	±7.5%
KBR67	Kenya	250	436.5 ± 9.91	2.3	-5.0401~3.3219	0	±7.5%
KRN94	Mexico	500	587.2 ± 7.38	1.3	-0.0249~0.0189	0	±5.0%
KBR77	Kenya	500	592.5 ± 8.30	1.4	-2.3122~2.4810	0	±5.0%
CBD128	United Kingdom	500	589.6 ± 5.93	1.0	-1.3569~1.7639	0	±5.0%
KAY42	China	500	593.2 ± 3.77	0.6	-1.0115~1.3823	0	±5.0%
SC12	India	500	589.9 ± 8.80	1.5	-3.9329~2.9836	0	±5.0%
KBR89	India	500	587.8 ± 23.99	4.1	-10.496~4.2361	3	±5.0%
CBD127	China	500	587.8 ± 23.57	4.0	-6.822~9.0167	8	±5.0%
KAN101	India	500	547.6 ± 29.57	5.4	-8.6591~7.1950	9	±5.0%

RSD: relative standard deviation; SD: standard deviation.

**Table 4:** Active pharmaceutical ingredient content results for amoxicillin dry oral suspension samples.

Sample code	Country of origin	Labelled active ingredients (mg/5 ml)	Chemical content as % label claim (RSD)	
			Day 0	Day 7
Control	United Kingdom	250	93.56 (0.26)	90.12(0.63)
KAN118	Kenya	125	98.56 (1.36)	96.13 (0.2)
KBR63	India	125	99.56 (0.25)	95.48 (0.67)
KBR82	India	125	101.18 (0.96)	96.70 (0.19)
KAY43	India	125	98.56 (0.68)	96.24 (0.52)
KBR83	Kenya	125	95.60 (1.12)	90.11 (0.64)
SC06	Kenya	125	104.21 (0.97)	100.08 (1.21)
ZIM106	Kenya	125	102.97 (0.77)	95.32 (0.26)
ZIM113	India	125	101.58 (0.33)	95.71 (0.36)
KAY54	United Arab Emirates	250	100.22 (0.21)	97.03 (1.35)
SB38	Kenya	250	103.91 (1.05)	96.79 (0.33)
SB19	Egypt	125	92.81 (1.00)	**88.27 (1.73)**
SC03	India	125	91.30 (1.57)	**88.24 (0.57)**
KBR74	India	125	**86.02 (0.53)**	**81.20 (1.07)**

RSD: relative standard deviation; USP limits: at 90%-120% of label claim.

**Table 5:** Active pharmaceutical ingredient content results for amoxicillin/clavulanic acid dry oral suspension samples.

Sample code	Country of origin	Labelled active ingredients (mg/5 ml)	Chemical content as % label claim for clavulanic acid (RSD)		Chemical content as % label claim for amoxicillin (RSD)	
			Day 0	Day 7	Day 0	Day 7
KAN119	India	228	112.73(1.78)	100.01(0.84)	108.23(0.94)	102.00(0.20)
KBR60	United Arab Emirates	228	113.48 (0.47)	102.20 (0.58)	104.22(0.27)	100.04(0.37)
KRN93	Canada	457	98.99 (0.83)	90.62(0.48)	102.24(0.33)	96.20 (0.66)
SC05	United Kingdom	228	99.51 (0.93)	96.18 (0.70)	96.53 (0.68)	90.53 (0.65)
WST98	India	228	101.03(0.23)	96.67 (0.48)	91.66 (1.83)	86.83 (0.80)
KBR80	India	228	105.49 (1.29)	98.34 (0.24)	92.02 (1.25)	88.85 (1.95)
CBD132	India	228	116.93(0.64)	114.64 (0.73)	92.54 (1.33)	89.12 (1.97)
SC011	France	642.9	96.50(0.75)	88.91 (0.58)	96.90 (0.57)	90.75 (1.15)
KAY40	Jordan	642.9	94.43 (0.69)	86.35 (0.87)	94.29 (0.22)	91.75 (0.77)
SC08	India	228	101.70 (0.28)	96.61 (0.21)	82.00 (0.93)	80.22 (0.30)
CBD137	India	457	94.03 (1.00)	91.30 (1.77)	89.76(0.90)	83.22 (1.03)
CBD133	India	228	93.43 (0.8)	89.93 (0.67)	85.12 (1.63)	82.07 (0.57)

RSD: relative standard deviation; USP limits: amoxicillin at ~90%-120% and clavulanic acid at 90%-125% of label claim.

**Table 6:** Active pharmaceutical ingredient and dissolution test results for amoxicillin capsules.

Sample code	Country of origin	Labelled active ingredients (mg)	Chemical content as % label claim (RSD)	Dissolution chemical content as % label claim (RSD)
KAY52	China	250	95.91 (0.71)	99 (3.71)
SC10	Kenya	250	98.24 (0.26)	99 (1.33)
SB32	India	250	93.93 (0.27)	97 (4.51)
KAY56	India	500	97.08 (0.34)	97.8 (5.3)
KAY51	India	250	98.60 (0.66)	95 (1.8)
ZIM109	Kenya	250	95.98 (0.29)	94 (5.3)
CBD138	Kenya	250	98.12 (1.92)	93 (3.67)
KBR78	China	250	94.09 (1.12)	92 (1.7)
KAN120	United Kingdom	500	96.67 (0.63)	100 (2.52)
KBR87	India	250	95.06 (0.23)	91.1(5.8)
SC013	India	500	94.20 (0.95)	90.70 (4.30)
SB021	China	500	92.60 (1.64)	88 (1.79)
KBR67	Kenya	250	90.74 (0.55)	88(5.3)
KBR84	Kenya	500	94.34 (1.99)	82.1 (3.80)
KRN94	Mexico	500	91.94 (0.54)	82 (5.2)
KBR77	Kenya	500	94.78 (0.11)	82.10 (8.3)
CBD128	United Kingdom	500	96.57 (0.26)	81.4(4.6)
KAY42	China	500	94.89 (0.62)	**76.4(7.5)**
SC12	India	500	93.91 (0.46)	**75.5 (7.5)**

RSD: relative standard deviation; USP limits: content determination at ~90%-120% and dissolution not less than (NLT) ~80% of label claim.

**Table 7:** Content determination and dissolution test results for amoxicillin/clavulanic acid tablets.

Sample code	Country of origin	Labelled active ingredients (mg)	Chemical content as % label claim (RSD)		Dissolution chemical content as % label claim (RSD)	
			Clavulanic acid	Amoxicillin	Clavulanic acid	Amoxicillin
Control	United Kingdom	625	94.00 (0.99)	95.00 (1.46)	101 (1.44)	94.00 (0.76)
KAN95	India	375	99.82 (0.59)	93.13 (0.29)	88.70 (3.40)	97.40 (2.80)
SB36	India	375	115.57 (1.37)	87.81 (1.24)	ND	ND
CBD131	India	625	105.81 (0.35)	99.84 (0.39)	77.60 (7.90)	88.10 (8.6)
KBR90	India	625	97.80 (0.50)	93.40 (0.44)	75.2 (1.61)	77.50 (1.97)
SB57	India	625	98.04 (1.19)	92.13 (0.31)	88.0 (4.80)	87.60 (4.20)
CBD136	India	625	113.00 (1.28)	84.81 (1.60)	ND	ND

RSD: relative standard deviation; USP limits: content determination for amoxicillin at ~90%-120% and clavulanic acid at 90%-125% of label claim. Dissolution: NLT 85% for amoxicillin and NLT 80% for clavulanic acid of label claim; ND: not done.

**Table 8:** Summary of content determination and dissolution test for samples from the same manufacturers but differing in quality.

Sample code	Origin	Sample type	Labelled active ingredients (mg)	Summary	
				Potency determination	Dissolution test
KAY56	India	Capsule	500	Complied	Complied
KAY43		Amoxicillin oral suspension	125	Complied	N/A
KAN95		Amoxicillin/clavulanic acid tablets	375	Complied	Complied
CBD133		Amoxicillin/clavulanic acid oral suspension	228	Failed	N/A

KBR63	India	Amoxicillin oral suspension	125	Complied	N/A
SC08		Amoxicillin/clavulanic acid oral suspension	228	Failed	N/A

KAY42	China	Capsule	500	Complied	Failed
KAY52		Capsule	250	Complied	Complied

KRN93	Canada	Amoxicillin/clavulanic acid oral suspension	457	Complied	N/A
SC03	India	Amoxicillin oral suspension	125	Failed	N/A
CBD128	New Zealand	Capsule	500	Complied	Complied
SC13	France	Capsule	500	Complied	Complied
KRN94	Mexico	Capsule	500	Complied	Complied

N/A: not applicable.

## Data Availability

The authors confirm that the data supporting the findings of this study are included within this published article.
